# Cost-effectiveness analysis of robot-assisted gait training in patients with bilateral spastic cerebral palsy

**DOI:** 10.1186/s12962-023-00475-3

**Published:** 2023-09-11

**Authors:** Stanislava Klobucká, Robert Klobucký, Katarína Valovičová, Pavol Šiarnik, Branislav Kollár

**Affiliations:** 1 Rehabilitation Centre Harmony, Bratislava, Slovakia; 2 Institute for Sociology, Slovak Academy of Sciences, Bratislava, Slovakia; 3 1st Department of Neurology, Faculty of Medicine, Comenius University Bratislava, Bratislava, Slovakia; 4 Faculty of Medicine, Slovak Medical University, Bratislava, Slovakia

**Keywords:** Neurorehabilitation, Health technology assessment, Advanced rehabilitation technologies, Lokomat^®^, Cerebral palsy, Gross motor functions

## Abstract

**Background:**

To date, there have been no published studies evaluating the cost-effectiveness of robot-assisted gait training (RAGT) in adolescent and adult patients with cerebral palsy (CP). The study´s aim was to analyse the cost-effectiveness of RAGT versus conventional kinesiotherapy (CON) from the health care provider’s perspective.

**Methods:**

We expressed the cost-effectiveness of RAGT in the Lokomat^®^ system after analysing the costs and effects of RAGT and conventional therapy through the Incremental Cost-Effectiveness Ratio (ICER) based on a bicentric randomized controlled study, in which we demonstrated that the intensive RAGT regimen is more effective than conventional therapy in terms of improvements in gross motor functions in adolescent and adult patients with bilateral spastic CP.

**Results:**

According to the calculated ICER ratio for Lokomat^®^, an additional improvement per unit of effect (1% in GMFM), compared to conventional therapy, results in an average cost increase of EUR70.38 per patient in a therapeutic block consisting of 20 TUs (Therapeutic Units).

**Conclusion:**

However, from the comprehensive analysis of the results and evaluation of the long-term effects, it follows that RAGT applied in adolescent and adult patients with bilateral spastic CP is not only more effective in terms of evaluation of monitored clinical parameters, but in the long term it is also more cost-effective compared to conventional therapy.

## Background

In the last decade, there has been a noticeable increase in the use of robot-assisted rehabilitation devices, particularly in patients after stroke, cerebrospinal trauma and, last but not least, in children with CP [1, 2]. These technologies allow intensive repetitive targeted training stimulating neuroplasticity and have proven themselves not only as a therapeutic tool, but also as an exact evaluation tool [1]. Advanced rehabilitation technologies are expensive and therefore their availability as part of standard rehabilitation care is limited not only in Slovakia.

Studies on the economic sustainability of robotic technologies for rehabilitation are published very sporadically in scientific journals. In fact, it is easier to prove clinical effectiveness than cost-effectiveness and the sustainability of innovative technology in the short, medium and long term [2].

To date, there have been no published studies evaluating the cost-effectiveness of RAGT in adolescent and adult patients with CP. The variability of CP manifestations in individual patients is diverse [3]. In the individual forms of CP, the severity of the disability varies from mild disorders to severely disabling conditions that exclude the affected individual from society, which makes it impossible to generalise about the cost of therapeutic and nursing care for these patients.

One of the basic methods of economic analysis in healthcare is Cost-Effectiveness Analysis (CEA), which can be part of the evaluation of the effectiveness of new technology by objectifying the consequences of the use of allocated resources [4].

The aim of this work, through a bicentric randomized controlled study, is to analyse the cost-effectiveness of robot-assisted locomotion therapy in the Lokomat® system versus conventional rehabilitation in adolescent and young adult patients with CP from the health care provider’s perspective.

## Materials and methods

**Fig. 1:**
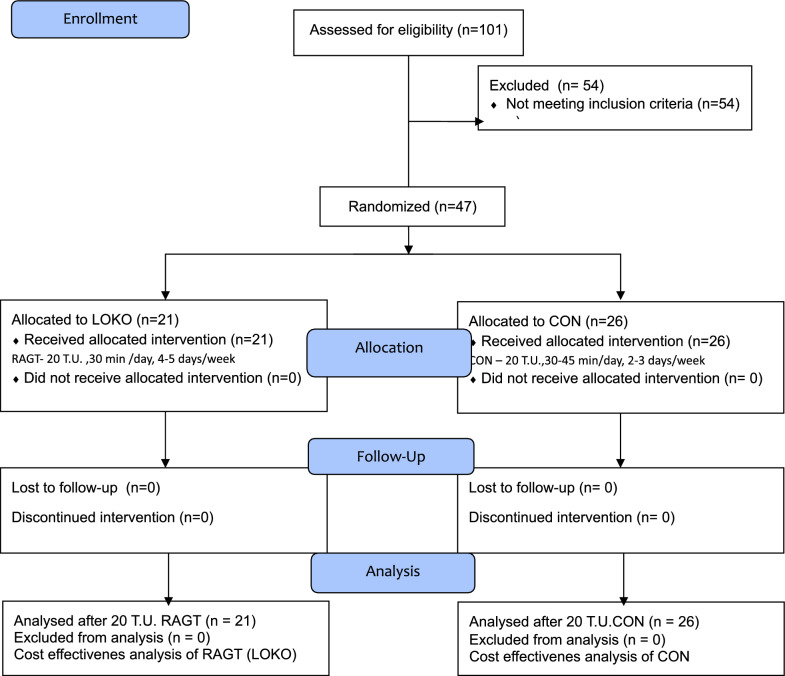
Flowchart of participants’ enrollment and randomization

The cost-effectiveness analysis was processed on the basis of our data from a published study that took place in two outpatient medical rehabilitation facilities in Slovakia in the period from September 2009 until August 2018 [5]. 47 adolescent and adult patients with a bilateral spastic form of CP were included in the study (Table 1). The first group (LOKO) consisted of 21 patients of the rehabilitation centre, which offers the possibility of robot-assisted rehabilitation; these patients underwent 20 TUs of RAGT. The second group (CON) consisted of 26 patients who underwent 20 TUs of conventional kinesiotherapy in another rehabilitation centre in which a RAGT device was not available (Fig. 1).

All subjects – patients, parents or legal representatives of patients were informed about the course and conditions related to the applied therapy, as well as about the use of test results for research purposes, and they gave the informed consent before inclusion. This study was conducted in accordance with the principles of the Declaration of Helsinki and Good Clinical Practice (GCP) and was approved by the local ethics committee.

### Therapeutic intervention

**Fig. 2:**
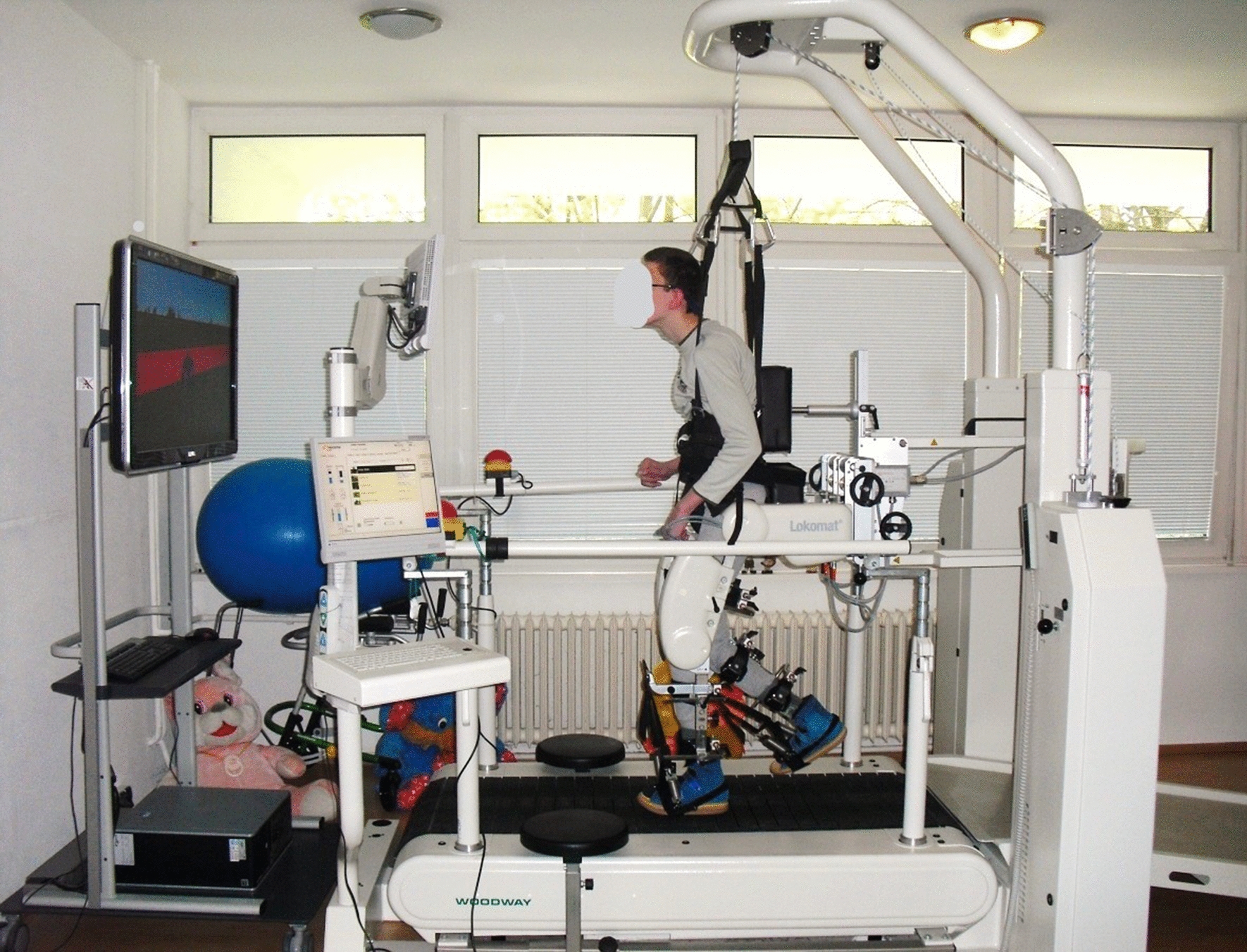
Robot-assisted locomotion therapy in a virtual reality-based environment in the Lokomat^®^

Patients from Group 1 (LOKO – an experimental group) underwent 20 TUs of robot-assisted locomotion therapy in a virtual reality-based environment in the Lokomat® system (Hocoma Inc., Volketswil, Switzerland), which we classify as exoskeletons (a concept based on the patient’s interaction with electronically controlled orthoses, e.g. while walking on a treadmill) (Fig. 2). Patients completed a therapeutic block within 4–6 weeks with a frequency of 3–5 times a week. During this period, robot-assisted locomotor therapy using the Lokomat® system was the sole therapeutic intervention. Patients in this group underwent RAGT for the first time, and no other kinesiotherapy was scheduled.

One therapeutic unit of RAGT lasted for 55 min, including the adjustment and positioning of the patient in the device (approximately 15 min), the walking session (30 min), and the removal/uninstallation of the device after the intervention (approximately 10 min), resulting in a total duration of 55 min per TU. [5].

Patients from Group 2 (CON – a control group, conventional therapy) underwent 20 TUs of individual kinesiotherapy 2–3 times a week during 7–10 weeks under the supervision of a physical therapist. One TU lasted for 30–45 min. The therapeutic procedure in this study represented usual care in clinical practice. In the current understanding, traditional or conventional rehabilitation procedures are those that are carried out exclusively by the physical therapist [6]. Individual exercises were focused on improving motor control, sitting and standing stability, gait, and activities of daily living (ADL).

In Slovakia, the most commonly used methods include Vojta’s method of reflex locomotion, neurodevelopmental treatment (NDT – Bobath concept), occupational therapy (ergotherapy), physical therapy (e.g., magnet therapy, bio laser phototherapy), and some patients underwent complementary methods of therapeutic rehabilitation – synergetic reflex therapy, ball training, circular training, TheraSuit, hydrotherapy, etc. – in various combinations and at different frequencies depending on the type and options of the healthcare facility they have been attending [5].

Despite consistent efforts to standardize therapeutic interventions, we made certain adaptations to some extent to the individual capabilities of patients and therapists, particularly regarding the duration and frequency of the therapeutic sessions, as conventional therapy was applied in a different rehabilitation centre.

### Evaluation of motor functions

All clinical evaluations and examinations were performed within 24 h before the therapy and within 24 h after the last TU. We determined the severity of disability using the GMFCS classification system (Gross Motor Function Classification System), which evaluates motor functions with a particular emphasis on sitting, walking and locomotion (also using a wheelchair), taking into account the age of the patient with CP [7]. Subsequently, patients were tested using the Gross Motor Function Measure (GMFM-88) [8, 9].

Walking time, distance and average walking speed during each training session was evaluated by the Lokomat® system.

### Cost analysis

For both types of therapy, we calculated the following:

- Hourly costs for one physical therapist (€/h).
- Average number of physical therapists needed for one TU for one patient.
- Average duration of one TU (min).
- Average number of TUs during the study period.
- The price per one TU.

In the costs of RAGT, we include the cost of purchasing the robotic device, the number of years until the amortisation of the robotic device, and the annual costs of routine maintenance (service costs). Based on the above, we were able to calculate the hourly price of the therapeutic intervention (which in our case corresponds to 1 TU) and the total price of the therapeutic block consisting of 20 TUs.

### Cost-effectiveness analysis

To evaluate the cost-effectiveness of RAGT in the Lokomat^®^ system, a cost-effectiveness analysis using the Incremental Cost-Effectiveness Ratio (ICER) was performed in this study.

The costs that are included in the calculation of the analysis are monitored from the health care provider’s perspective.

### Statistical analysis

We processed data using MS Office Excel and SPSS 21.0 for Windows. To process the obtained data, we used descriptive statistics, non-parametric statistics, confidence interval and effect size. Data for cost calculation were collected by the bottom-up (micro-costing) method alongside standard patient care.

The two groups were compared at the baseline using χ2 test/Fisher´s exact test for categorical variables and the independent sample t-test (two-tailed, significance level p < 0.05) for continuous variables. Since the data did not follow a normal distribution across all datasets, we employed the non-parametric Wilcoxon test for paired values to compare the input and output GMFM values in each group. To evaluate inter-group differences in the percentage improvement in GMFM (RAGT vs. CON), we used the MannWhitney test for two independent datasets. Results were considered statistically significant at p < 0.05 and highly statistically significant at p < 0.001. To determine the substantive/practical significance of the differences, we calculated Cohen´s effect size (d) using the pooled standard deviation. According to the criteria proposed by Cohen, the coefficient d = 0.01–0.2 is interpreted as a very small effect size, d = 0.2–0.5 as a small effect size, and d = 0.5–0.8 as a medium-sized effect causing the difference. Values of d = 0.8–1.2 represent a large effect size, and d > 1.2 indicate a very large effect size. The calculation of mean improvements and the Cohen´s coefficient d was completed by computing the 95% confidence interval (CI 95%).

## Results

47 patients (27 male and 20 female) with bilateral spastic CP aged 15.1–35.1 years (mean age 21.2 (SD ± 5.33)) underwent 20 TUs according to the scheduled protocol. Baseline data on all patients included in the study are shown in Table 1.

**Table 1:** Patient characteristics

	LOKO group (n = 21)	CON group (n = 26)	P
Mean (± SD) age (years)	18.3 (± 3.84)	23.4 (± 5.33)	0.482
Range, minimum-maximum age (years)	15.1–27.0	15.1–35.1	
Gender n (%)			
Female	10 (47.6%)	10 (38.5%)	p = 0.528
Male	11 (52.4%)	16 (61.5%)	
GMFCS level n (%)			
I	1 (4.8%)	0	p = 0.835 Z = −0.209
II	3 (14.3%)	4 (15.4%)	
III	9 (42.9%)	12 (46.2%)	
IV	8 (38.1%)	10 (38.5%)	

*LOKO* experimental group, *CON* control group, *GMFCS* gross motor function classification system

p - level of statistical significance, significant at p < 0.05

### Comparison of mean improvements in motor functions in the RAGT vs. CON groups

21 patients (LOKO group) underwent 20 TUs of RAGT in the Lokomat^®^ system. We observed a statistically significant improvement in motor functions (p < 0.001), which represented the small Cohen’s effect size (d) in dimensions B, C, D, E, GMFM total score, and the medium-sized Cohen’s d effect size in GMFM dimension A [5].

26 patients (CON group) underwent 20 TUs of individual kinesiotherapy under the supervision of a physical therapist. A statistically non-significant change in the evaluation of motor functions in patients after conventional kinesiotherapy represented a very small effect size in the evaluated GMFM parameters.

Comparing the mean improvements (%) of endpoints after 20 TUs in both groups (LOKO vs. CON), we documented a statistically significant difference (at a level of statistical significance of 0.000) in all dimensions A–E, as well as in overall GMFM improvement in favour of the LOKO group. By calculating the Cohen’s effect size (d), we found that the statistical significance is accompanied by a substantive significance in all dimensions, and represented a very large effect size in all compared items [5] (Table 2).

**Table 2:** Comparison of improvement/change in motor functions using GMFM-88 total score between LOKO and CON groups after 20 Tus

	Mean improvement in %±SD (CI 95%)		Z	P	d CI 95%
	LOKO group (n = 21)	CON group (n = 26)			
Total GMFM	9.43 ± 5.73 (6.989–11.891)	0.80 ± 1.68 (0.154–1.446)	−5.590	0.000	2.147 (1.426–2.867)

Values are presented as mean ± SD

*GMFM* gross motor function measure

d – Cohen’s effect size, rate of substantive significance of differences

d = 0.2–0.5 (small difference), d = 0.50–0.80 (medium-sized difference), d = 0.80–1.2 (large difference), d > 1.2 (very large difference)

p – statistical significance level, significant at p < 0.05

Z – value of the test criterion (statistical calculation) was obtained using the Mann-Whitney U test

CI 95% – confidence interval

### Cost analysis

We bought the Lokomat^®^ in the Rehabilitation Centre Harmony in 2007. Its purchase price was €321,160.34 (SKK 12 million at the time). For comparison, the price of the newest version of Lokomat^®^ increased to €881,292 in 2022.

According to the classification in depreciation groups, the amortisation period of Lokomat^®^ was determined to be 6 years. Service inspections, including safety and technical inspections including personnel costs and spare parts, are carried out once a year by the supplying (authorised) company. Extra repairs out of scope of the regular safety and technical inspection are not paid for separately and are already included in the flat rate price. Due to the significant increase in the prices of advanced rehabilitation technologies, the prices for the year 2022 are also presented (Table 3).

**Table 3:** Purchase price of Lokomat^®^, accounting depreciation, service and maintenance costs of Lokomat^®^

Medical instrument	Procurement price / €	Monthly costs for accounting depreciation / €	Annual costs for accounting depreciation / €	Monthly service costs / €	Annual service costs / €
Lokomat^®^ (2007)	321,160.34	4,460	53,527	671.7	8,060
Lokomat^®^ for FreeD Kombi (2022)	881, 292	12, 240	146, 882	1,700	20,400

### Total costs of robot-assisted gait training vs. conventional kinesiotherapy

The direct costs of RAGT are characterised in Table 4. This is a calculation of the annual costs, from which the costs per one TU in 2013 are calculated. Conventional kinesiotherapy was chosen as a comparative/alternative method of therapy.

Both therapeutic methods have approximately the same requirements for the size of the space; we have included the cost of this space in the calculation of the price per one TU.

Costs are presented in an annual breakdown/summary as well as for individual therapy (one TU). In the case of RAGT, using a total of 1,440 h per year, this represents approximately 6 working hours per day, 5 days per week.

According to the annual work calendar, 250 working days are included. The average number of working hours per year per month is 173.9 h.

The resulting amount of personnel expenses is the sum of not only wage costs (gross wage), but also the costs of employer’s contributions, health and social insurance in the amount of 35.2% of gross wage. Wage costs were comparable in both groups throughout the entire period. The average hourly price of a physical therapist’s work was calculated from wage costs for 2013 and, for comparison, also for 2022 (Table 4). The number of physical therapists required to perform therapy using Lokomat^®^ and conventional kinesiotherapy depends on their physical fitness and the degree of a patient’s disability. In general, it can be said that in practice one to three physical therapists are used within one TU of conventional therapy in patients with CP, depending on the severity of their disability. For more severely disabled patients (GMFCS III–V), the presence of at least 2 physical therapists is necessary when verticalising them in a conventional manner. However, during therapy using the Lokomat^®^ system in a more seriously disabled patient, the physical therapist can also operate other advanced rehabilitation technologies at the same time, so in reality, often 0.5 physical therapists are needed. In view of the above, for the calculation of personnel costs in the intervention group (LOKO), we included the price of the work of 1 physical therapist for 1 h (1 TU), and in the case of conventional therapy (CON) the price of the work of 2 physical therapists for 1 h (1 TU).

**Table 4:** Costs of LOKO and CON therapeutic interventions per patient in 2013 and 2022

	LOKO 2013	LOKO 2022	CON 2013	CON 2022
Average annual personal costs €/year	17,861	23,168	2 × 17,861	2 × 23,168
Consumables €/year	548	1,045	548	1,045
Price of the work of one physical therapist €/hour	8.56	11.1	8.56	11.1
Number of TUs /year	1,440	1,440	1,440	1,440
Energy (gym) €/month €/year	250 3,000	500 6,000	250 3,000	500 6,000
Administrative expenses €/year	128	143	128	143
Depreciation €/month €/year	4,460 53,527	12, 240 146,882	–	–
Service costs €/year	8,060	20,400	–	–
Price per 1 TU /€	57.73	137.25	27.36	37.17
Total cost of therapy (20 TUs) €/month €/year	6,927 83,124	16,470 197,638	3,283.2 39,398	4,460.3 53,524

We calculated the annual cost of therapy as the sum of individual items; in conventional therapy, we added the cost of work for 1 physical therapist to the calculation of annual costs (this means double the average annual personal costs).

We then calculated the price of one TU of individual therapies (LOKO and CON) as a proportion of annual costs and the number of therapeutic hours per year. From this price we calculated the total cost of a therapeutic block consisting of 20 TUs.

When comparing the direct costs of therapy in the Lokomat^®^ system with conventional kinesiotherapy, it is obvious that conventional kinesiotherapy is 53–73% cheaper (Table 4).

### Cost-effectiveness analysis

CEA (Cost-Effectiveness Analysis) measures the cost per unit of effect. As intervention costs (RAGT, LOKO) are higher than standard care costs (conventional, CON), we evaluated the ratio of incremental costs and ICER effects.

ICER = C1–C2/E1–E2 is defined as the ratio of the difference in costs of specific therapeutic interventions and the difference in clinical effects, where C1 and C2 are the costs of the entire rehabilitation process (20 TUs) for RAGT and conventional therapy, E1 and E2 document the effectiveness of each therapy in terms of the primary outcome.

In our case, we will divide the difference in the cost of therapy consisting of 20 TUs in the Lokomat^®^ system and the cost of conventional therapy (20 TUs) by the difference in the improvement/change of motor functions after completion of individual therapeutic interventions.

$${\text{ICER}}\,{\text{ = }}\,{\text{C LOKO}}\, - \,{\text{C}}\,{\text{CON}}/{\text{E}}\,{\text{LOKO}}\, - \,{\text{E}}\,{\text{CON }}$$

The result is then interpreted as the cost per patient with CP per unit of improvement in the GMFM test when switching/changing from conventional therapy to RAGT (interventional). In general, it can be concluded that the lower the ICER, the higher the value for the costs incurred.

We found that the average cost of a 1% improvement in GMFM in one therapeutic block consisting of 20 TUs of therapy in Lokomat^®^ per one patient is €122.43. The average cost of a 1% improvement in GMFM in one therapeutic block consisting of 20 TUs of conventional therapy is €684. According to the calculated ICER ratio for Lokomat^®^, an additional improvement per unit of effect (1% in GMFM) versus conventional therapy (more cost-effective), results in an average cost increase of €70.38 per patient in a therapeutic block consisting of 20 TUs (Table 5).

In Table 5, we also present the results for the year 2022 for the potential purchase of a new Lokomat^®^ model (Lokomat^®^ Pro FreeD Kombi), but the results are only an extrapolation from the analysed data of the older Lokomat^®^ model. Lokomat^®^ Pro FreeD is a more advanced version of robot-assisted gait training with a robot-guided pelvis, but we cannot guarantee identical results; rather we expect a significant improvement in motor functions.

**Table 5:** Cost-effectiveness analysis of RAGT compared to conventional kinesiotherapy

	Mean improvement in GMFM after 20 TUs in %	ICER LOKO vs. CON per 1 TU (2013, 2022)	ICER LOKO vs. CON per 20 TUs (2013, 2022)	Years	Price/costs of 1 TU of LOKO in €	Price/costs of 20 TUs LOKO in €	C/E per 1 TU of LOKO/CON	C/E per 20 TUs of LOKO/CON
Lokomat^®^	9.43	3.52 11.59	70.38 231.94	2013	57.73	1,154.6	6.12	122.43
				2022	137.25	2,745	14.53	291.09
Conventional kinesiotherapy	0.8			2013	27.36	547.2	34.2	684
				2022	37.17	743.4	46.46	929.25

The ratio of costs to the achieved therapeutic effect (C/E) is lower with RAGT. With higher initial costs for RAGT, significantly higher clinical effectiveness is achieved compared to conventional therapy, which has lower costs but also lower clinical effectiveness (C LOKO/E LOKO < C CON/E CON). The cost of the achieved mean improvement (C/E) in one therapeutic block is higher with conventional therapy versus Lokomat® in 2013 as well as in 2022.

Thus, RAGT appears to be a more cost-effective alternative in the long term.

## Discussion

Despite advancements in the treatment and care of patients with CP, a certain level of disability still persists in many cases, therefore, it remains imperative to continuously search for and develop further therapeutic procedures and methods to enhance their health and quality of life.

A general prerequisite for satisfactory rehabilitation results is its timely initiation, high intensity, correct timing, and targeted functional kinesiotherapy focused on the performance of specific tasks (task-oriented training). Robotic devices that allow targeted repetitive movements, i.e., therapy can be more intensive and potentially more effective, help to meet these criteria. Despite the proven clinical effectiveness, the economic aspect is a significant obstacle to their wider use, because advanced rehabilitation technologies are very expensive and insurance companies usually do not reimburse them. However, the sums for such therapy requested from private providers are often liquidating for the families with children suffering from medical disabilities. There is a risk that they will not rehabilitate at all or significantly insufficiently, thereby secondary burdening the system with even more complications that will require significantly higher costs.

The aim of this study was to analyse the clinical and cost-effectiveness of both therapeutic approaches from the perspective of a health care provider based on the objectification of RAGT’s impact on motor functions in adolescent and adult patients with bilateral spastic CP compared to conventional kinesiotherapy.

### Evaluation of clinical effectiveness

In accordance with the works of foreign authors, we have confirmed that RAGT has a positive effect on the motor functions of patients with CP [10, 11]. A meta-analysis of studies suggests that gait training has a more significant effect on motor function than conventional rehabilitation [12]. In our study evaluating the effect of RAGT vs. conventional kinesiotherapy, we observed a significant improvement in motor functions (p < 0.001), evaluated using all five GMFM-88 dimensions in adolescent and adult patients with CP [5]. The results are also remarkable for the reason that – according to predictive indicators – further improvement in this age category was no longer expected [13, 14]. By comparing the mean improvements in the evaluated parameters of gross motor functions (GMFM-88) after 20 TUs in two treatment groups, we observed a significantly (p < 0.001, Cohen’s d = 2.147) greater improvement in the experimental RAGT group with intensive regimen compared to the control group consisting of patients who underwent conventional training. Furthermore, such improvement has also persisted after 3–4 months [5].

### Cost analysis

Robot-assisted technologies require high investments, and their maintenance and normal operation are relatively expensive depending on the type of rehabilitation, which is usually the main argument against their incorporation into the therapeutic process [2].

Analyses evaluating new technologies are a relatively new topic compared to drug studies. There is still relatively little scientific evidence and attempts to obtain information are difficult, but the need for evaluation of medical technologies is crucial to support the decision-making process. Studies published so far show that patients who rehabilitate with robotic support improve faster than those who are treated conventionally [12, 15–18].

In their meta-analysis, Carpino et al. [19] demonstrated that robot-assisted gait training allowed more patients after stroke to regain walking independence compared to conventional therapy, and from an economic point of view robot-assisted training is a more sustainable method despite the difference between the costs of the individual therapeutic procedures compared. Similarly, Pinto et al. [20] confirmed in the Budget Impact Analysis of their study that gait training through exoskeletons is associated with lower costs in patients with spinal cord injury compared to conventional therapy.

Also in our randomized clinical study, we demonstrated by cost analysis that RAGT appears to be more effective in the long term versus conventional therapy. Using the calculation of the ICER, we found that in order to obtain an additional unit of improvement (1% in the GMFM test) by switching from conventional to robotic therapy at the time of the study (2009–2018), an increase in costs of €70.38 for the entire therapeutic block consisting of 20 TUs would be necessary. Very similar results were also reached by Carpino et al. [19], who found that robot-assisted gait training in post-stroke patients is approximately €62.36 more expensive compared to conventional therapy to achieve an increase in patient walking speed of 1 m/s. If we consider the total cost, we cannot say that it is a high amount. However, the decision still remains on the side of health care payers and their willingness to pay for this intervention. The difference between the initial cost of RAGT and conventional therapy is considerable, but the initial cost decreases with the number of working hours and years of possible use of the device.

### Who will pay for robot-assisted rehabilitation?

In Slovakia, the use of advanced rehabilitation technologies is centralised in the National Rehabilitation Centre and specialised hospitals, where this therapy is reimbursed by the insurance company as part of the “bed-day”. The price of a day of treatment was around €120–140 at the time of this analysis. At that time, the price of a reduced treatment day in rehabilitation centres (including our Harmony Rehabilitation Centre) was around €15 for a long time – more than 10 years. In addition, insurance companies regularly refuse to pay for robot-assisted therapy in an inpatient setting. However, due to the financial undersizing of rehabilitation clinics, inpatient wards and inpatient settings, there are long-term negotiations on increasing payments to workplaces that meet the criteria of neurorehabilitation workplaces.

Currently, there is an increasing number of workplaces in Slovakia where the patient pays extra for this therapy or is forced to pay for it in full him-/herself.

In many European and American countries, we observe a different approach in acquiring, financing, and using advanced rehabilitation technologies adapted to economic conditions and health needs [2]. Rehabilitation with the help of robot-assisted systems is usually included in the reimbursement system, but in most cases the financial participation of the patient is taken into account.

According to the International Covenant on Economic, Social and Cultural Rights (ICESCR), ‘States Parties to the present Covenant recognise the right of everyone to the enjoyment of the highest attainable standard of physical and mental health’. Among other things, it also declares ‘the legal right to a health system that enables all people equally to achieve the highest attainable standard of health (for them personally)’. Although this fact does not create a directly enforceable right of an individual patient to claim reimbursement, it requires national health systems to provide sufficient rehabilitation services (including robot-assisted rehabilitation) and grant patients access to state of the art therapies [2].

Currently, the lack of rehabilitation centres specialised in paediatric patients, which would provide comprehensive rehabilitation paid from public resources, is deepening in Slovakia. With a lack of payments from health insurance companies, they are in real danger of disappearing, and patients will thus be dependent on private workplaces, where they often have to pay for healthcare in full.

Also debatable is the question of the necessary human resources for specific therapies. The literature reports that 1.19 physical therapists are needed to perform conventional therapy, while only 1 physical therapist is needed for RAGT, regardless of the type of robotic device [19]. These data are in contrast with other published data. Morrison [21] reported that 1 physical therapist is needed for RAGT, while up to 4 physical therapists are needed for manual-assisted gait training. This difference is likely due to the fact that Morrison compared intensive repetitive robot-assisted locomotion training with similarly intensive, but manually assisted training. Esquenazi et al. [22] similarly compared manually assisted gait training with robot-assisted gait training and concluded that one physical therapist is needed for therapy in the Lokomat^®^ system, while more than one physical therapist is needed for manually assisted training, especially in more severely disabled patients. Robot-assisted training is often performed in groups at many of the world’s leading rehabilitation clinics. One of the great advantages of advanced rehabilitation technologies is that less direct supervision is required during training, which allows parallel therapeutic interventions to be carried out, thus increasing the number of therapeutic units that can be provided by a single physical therapist. Thanks to this fact, it is possible to provide longer and more intensive therapies without increased costs [23, 24].

When using advanced technologies, it is possible to partially refrain from the traditional ‘one on one’ approach and use their full potential in terms of prolonging the therapy and increasing the intensity for several patients at the same time. This will make it possible to improve results without increasing costs, which will also increase financial effectiveness. However, robot-assisted rehabilitation should not be considered a substitute for a physical therapist, but as another means of making therapy more efficient [25, 26].

When evaluating the cost-effectiveness of advanced rehabilitation technologies, it might be worthwhile to modify the design of a unified standardised therapy with a predefined duration of therapy and to determine rather the ‘goals’ of the therapeutic intervention (adequate values of effectiveness) and then to assess the time in which patients reached these goals. This would make it possible to more realistically assess the costs of RAGT and CON, which also take into account rest days for patients when reaching the set ‘goals’, but also other aspects – non-medical direct costs (social services, home care, transport) and indirect costs (loss of working days of parents, caregivers, working patients etc.).

### Strengths and limitations of the study

This is the first study, to our knowledge, evaluating RAGT in adolescent and adult patients with cerebral palsy using of cost-effective analysis methods.

To date, only a few studies have evaluated the effects of RAGT on motor functions in adolescent and adult patients with CP.

In most studies concerning gait training in patients with CP, only dimensions D (standing) and E (walking, running, jumping) are assessed within the range of GMFM-66. However, after completing 20 TU of RAGT, we observed stabilization of axial muscle tone in our patients, resulting in improvements in sitting, crawling and rolling. Therefore, to objectively measure these observations, we decided to evaluate all GMFM-88 dimensions (A, B, C, D, E and total score) for the patients in this study.

The patient group was heterogeneous in terms of severity of disability, reflecting the standard population of the neurorehabilitation clinic.

In the study, we did not include children under the age of 15. Hanna et al. [13] analysed reference curves for motor development in individuals with CP, based on longitudinal observations across various age categories and different degrees of disability. According to these curves, there is an improvement of gross motor functions among individuals with CP at GMFCS level I until adolescence, and GMFCS level II until the age of 9–12 years. Subsequently, gross motor functions remain relatively stable, although they tend to decline at different ages depending on the severity of the disability. We deliberately selected patients who were not expected to exhibit motor improvement according to the developmental motor curves. The assessment of the clinical effectiveness of RAGT in children with CP has been the focal point of our previous studies [27, 28]. The inclusion of a cost-effectiveness analysis would certainly enhance the level of these investigations.

During the study, we attempted to conduct tests with the same physical therapists; however, due to organizational reasons, this was not possible in all cases.

Quality of life and ICF domains of personal or environmental factors were not assessed in detail. In the future, such an assessment could contribute to the analysis of the benefits of new interventions.

The study results provide new insights for the decision-making process regarding the utilisation of advanced rehabilitation technologies, aiming to support a functional, efficient, and high-quality healthcare system.

## Conclusion

The results of the presented analysis indicate that RAGT in an intensive regimen is more effective and, in the long term, more cost-effective in adolescent and young adult patients with CP compared to conventional therapy. There is also additional evidence that advanced rehabilitation technologies allow for more intense, longer-duration training that allows patients to regain or improve motor function compared to conventional rehabilitation. Therefore, from the point of view of the health care system, further research is needed regarding the cost-effectiveness of robotics in rehabilitation with larger cohorts of patients and with application in different diagnoses.

Ultimately, these facts can help in clinical practice when deciding on investments and choosing a therapeutic intervention.

## Data Availability

The dataset (s) supporting the conclusions of this article is(are) included within the article. Further information regarding the data can be obtained by contacting the corresponding author.

## Abbreviations

BS-CP: Bilateral Spastic Cerebral Palsy
CEA: Cost-Effectiveness Analysis
CP: Cerebral Palsy
GMFM: Gross Motor Function Measure
GMFCS: Gross Motor Function Classification System
ICER: Incremental Cost-Effectiveness Ratio
ICF: International Classification of Functioning, Disability and Health
LOKO: Lokomat group / RobotAssisted Gait Training
RAGT: RobotAssisted Gait Training
CON: Control group / Conventional therapy
TU: Therapeutic Unit
